# Biological depolymerization of lignin using laccase harvested from the autochthonous fungus *Schizophyllum commune* employing various production methods and its efficacy in augmenting in vitro digestibility in ruminants

**DOI:** 10.1038/s41598-022-15211-9

**Published:** 2022-07-01

**Authors:** Vidya Pradeep Kumar, Manpal Sridhar, Ramya Gopala Rao

**Affiliations:** National Institute of Animal Nutrition and Physiology, Adugodi, Bangalore, Karnataka 560 030 India

**Keywords:** Biochemistry, Microbiology

## Abstract

A laccase-producing hyper performer, *Schizophyllum commune,* a white-rot fungus, was evaluated for its ability to selectively degrade lignin of diverse crop residues in vitro. Relative analysis of crop residue treatment using laccase obtained from immobilized cells demonstrated degradation of 30–40% in finger millet straw and sorghum stover, 27–32% in paddy straw, 21% in wheat straw, and 26% in maize straw, while 20% lignin degradation was observed when purified and recombinant laccase was used. Further investigations into in vitro dry matter digestibility studies gave promising results recording digestibility of 54–59% in finger millet straw 33–36% in paddy straw and wheat straw, 16% in maize straw for laccase obtained from cell immobilization method, whereas 14% digestibility was observed when purified and recombinant laccase was used. Sorghum stover recorded digestibility of 13–15% across all straws treated with laccase. The results obtained elucidated the positive influence of laccase treatment on lignin degradation and in vitro dry matter digestibility. The present research gave encouraging figures confirming the production of laccase using the cell immobilization method to be an efficient production method commensurate with purified and recombinant laccase under conditions of submerged cultivation, proclaiming a cost-effective, environmentally safe green technology for effectual lignin depolymerization.

## Introduction

Ruminant livestock occupies a unique niche on account of their inherent ability to use crop residues as feeds, the key contributors to livestock feed resources as roughages, especially in the developing and transition countries. The widespread global availability of these residues and their importance in crop-livestock systems assign them the position of important strategic natural resources^1^. Considering the huge quantities of lignocellulosic biomass available and the high nutritive quality of their polymeric constituents, the hexose and pentose sugars, cereal straws normally contain at least 70% carbohydrates^2^ and are, therefore, no doubt a potential source of energy for livestock, which can be utilized through microbial fermentation in the rumen. Lignin has been reported as one of the most significant recalcitrance factors affecting biomass recalcitrance^3,4^ and the inability of rumen microbes to release adequate levels of energy from many crop residues has limited their value and utility for livestock production. Technologies that significantly enhance delignification and promote utilization of trapped energy from crop residues thus represent an unprecedented opportunity for enhanced productivity from existing agricultural systems, especially where crop residues are underutilized.

Among the various methods hitherto employed for improving nutrient availability from crop residues, biological treatment with white-rot fungi (Basidiomycetes) is known to be the safest green technology being endowed with the inherent capability to selectively degrade lignin on account of their major ligninolytic enzymes viz. Laccases, Lignin peroxidases, Manganese peroxidases, and Versatile peroxidases.

Laccase (benzenediol: oxygen oxidoreductase; EC 1.10.3.2) a blue-copper oxidoreductase is the most widely studied of these enzymes having application in a vast number of biotechnological processes, including materials science, bioremediation, biofuels, fibre modified nanobiotechnology, biosensor, food chemistry, paper, and pulp industry, and biodegradation^5–7^. Laccase catalyzes the oxidation of a wide range of substrates including phenolic compounds with the concomitant reduction of molecular oxygen to water^8,9^. Traditionally, laccase has been produced by white-rot fungi using submerged fermentation batch culture a very convenient nevertheless expensive process, especially if the enzyme requires a high fold purification as well as solid-state fermentation (SSF) from agro-waste materials^10–12^. The research to date about laccase production on agro-based residues was chiefly targeted toward biofuel and bioethanol production and laccase production at the laboratory level from fungi such as *Agaricus bisporus*,* Coriolus versicolor*,* Pleurotus ostreatus*,* Trametes versicolor*, and* Rhus vernicifera* has been widely reported^13^. *Schizophyllum commune,* a white-rot basidiomycete was studied for ligninolytic enzymes (manganese peroxidase, lignin peroxidase, and laccase) production in solid-state fermentation (SSF) for delignification of various agro-industrial residues from the banana stalk, corn cobs, sugarcane bagasse, and wheat straw, respectively, and were also employed for ethanol production from wood chips by consolidated bioprocessing^14^ while Han et al.^15^ used *S. commune* mixed with other species and reported that the fungal co-culture and the mixed lignocellulosic wastes contributed to the improvement of laccase activities and enhanced laccase yields within a short period.

WRF such as *Phanerochaete chrysosporium*,* Pleurotus* sp.,* Lentinus edodes*,* Coriolus versicolor*,* Phlebia* sp., and *Ceriporiopsis subvermispora* was used to ferment various crop residues like wheat straw, olive mill solid waste, madake bamboo, tanniniferous lespedeza plants, oil palm fronds, etc. which were then used for feeding ruminants as such or as crude enzyme extracts^16–20^. Microbial conversion though a practical and promising alternative for enhancing the nutritional value of crop residues results in severe losses in organic matter.

The amount of enzyme secreted by fungi in the native state is not sufficient to meet the current industry demands and created the dire necessity for methods of enhancing secretion or obtaining novel potent enzymes from wild isolates. Studies in this direction on the application of ligninolytic enzymes in general and laccase in particular to enhance the digestibility of crop residues for better ruminant productivity are scarce^21,22^. Laccase obtained from a wild isolate of the white-rot fungi *S. commune* showed positive delignification and enhanced digestibility for ruminants in vitro^23^. In the concerted efforts and in the light of this background the current study was conducted (a) to validate the potential of laccase enzyme harvested from NI-07 strain by various methods in delignification of crop residues and (b) to authenticate the efficacy of enzyme production encompassing greater efficiency, economic viability, reduction in production complexities and harnessing enormous stability to pave the future direction for utilization of the most economically feasible technology for bulk production of laccase to improve digestibility in ruminants.

## Materials and methods

### Chemicals

Unless otherwise stated, all chemicals used for analysis were of analytical grade and were purchased from Hi-Media. The substrate 2,2′-azino bis (3-ethylbenzthiazoline-6-sulfonic acid) (ABTS) was procured from Sigma–Aldrich (USA). Restriction enzymes and the *Pichia* GS115 strain were purchased from Invitrogen.

### Organisms

*Schizophyllum commune NI-07*, a potent laccase producer^23^ used in the present study, was isolated and deposited with MTCC, Chandigarh, and is available for procurement (MTCC 11893). *Trametes versicolor* (*MTCC* *138*) was used as the reference standard. Both cultures were maintained on Malt Extract Agar (MEA) slants and plates.

### Laccase production in liquid cultures

Laccase was produced under submerged cultivation conditions (SmF) using Malt extract broth (MEB) as the growth medium and modified basal salt solution (BSS) as the production medium^24^. Mycelial plugs (5 mm × 3/100 ml media) inoculated and cultured in MEB for 5 days (30 °C, 120 rpm) were used as inoculum for laccase production in BSS. One litre of BSS comprised of glucose (20 g), l-asparagine (2.5 g), l-phenylalanine (0.15 g), KH_2_PO_4_ (1 g), MgSO_4_·7H_2_O (0.5 g), CaCl_2_ (0.01 g), FeSO_4_·7H_2_O (0.01 g), MnSO_4_·4H_2_O (0.001 g), ZnSO_4_·7H_2_O (0.001 g), CuSO_4,_ 5H_2_O (0.002 g), and thiamine hydrochloride (1 mg). Flasks containing the production medium were incubated at 30 °C for 7 days (120 rpm). Cell-free culture supernatant was used as a crude enzyme for further analysis after centrifugation (2000×*g*, 15 min).

The medium used for laccase production under submerged conditions was optimized (SmF/Opt) using response surface methodology (RSM)^25^ and the Proc General Linear Model procedure of SAS (Version 9.3). This was adapted to maximize laccase production by altering the various culture conditions that affect the growth of fungal biomass by one factor at a time (OFAT) approach. Statistically optimized media obtained from RSM for enhancing laccase production contained 1.82% glucose, 1.35% fructose, 0.23% l-asparagine, 0.19% yeast extract, 2.45 mM *p*-anisidine and 1.77 mM catechol. Cultures were grown using similar conditions as mentioned above. Cell-free culture supernatant was used as a crude enzyme for further studies after centrifugation (2000×*g*, 15 min).

Whole-cell immobilization (Icell/Lac) was performed using inert matrix polyurethane foam (PUF). PUF sheets were procured from Shriram Polymers (Mumbai, India), cut into 1 × 1 × 1 cm cubes, pre-treated by boiling in water for 20 min at 80 °C, soaked overnight in methanol before washing with distilled water, dried, and autoclaved. Flasks containing 5 g/100 ml media of autoclaved cubes were inoculated with mycelial plugs (5 mm × 3/100 ml media) homogenized under aseptic conditions and incubated (30 °C) under continuous shaking (120 rpm) for five days. MEB used as growth media was decanted slowly from the flasks and replenished with BSS, retaining the cubes immobilized with fungus. Flasks were further incubated (30 °C, 120 rpm) for laccase production. Aliquots from the media were regularly sampled for measuring laccase activity, and laccase-rich media was harvested between the 6th and 7th days when there was maximum laccase production. Cell-free laccase-rich media was obtained by centrifugation (2000×*g*, 15 min) of the harvested media.

Purified laccase was obtained by subjecting the cell-free supernatant obtained using immobilization to 70% (NH_4_)_2_SO_4_ precipitation, kept overnight at 4 °C and centrifuged (5000×*g*, 30 min). The precipitate was dissolved in 0.4 M Na-acetate buffer (pH 5.2). The solution was dialyzed overnight against 0.1 M Na-phosphate buffer (pH 6.8) and loaded onto a Sephadex G-50 column (44 × 3 cm) equilibrated with the same buffer. Collected fractions were assayed for both protein and laccase activity. Active fractions were pooled and concentrated on an Amicon ultra cell-30 membrane, Millipore, USA (Pure/Lac) and reconstituted in sodium acetate buffer (pH 5.2) for further analysis.

The nucleotide sequence of the full-length cDNA from *Schizophyllum commune* containing a 1554 bp open reading frame encoding a polypeptide of 518 amino acid residues was adopted from Hatamoto^26^, and the gene was codon-optimized and directionally subcloned into the pPIC9K vector using EcoR1 and Not1 restriction enzymes. The vector was linearized using BglII and transformed into the *Pichia* GS115 strain^23^. Single colonies from the transformed plate were inoculated into 10 ml of buffered minimal glycerol (BMG) medium (prepared by using 100 ml of potassium phosphate buffer (1 M, pH 6.0), 100 ml 10× YNB, 2 ml 500× Biotin and 100 ml 10× glycerol and brought up to 1000 ml with sterile distilled water) and grown at 30 °C (250 rpm) for 18 h. The cells were then harvested by centrifugation (2000×*g*, 5 min). The supernatant was decanted, and the cell pellet was resuspended in buffered minimal methanol (BMM) (prepared using 100 ml of 10× methanol instead of glycerol). Other components are the same as BMG. Cultures were incubated at 30 °C (250 rpm), and 100% methanol was added to a final concentration of 0.5% (v/v) every 24 h to maintain induction. Cell-free culture media (Rec/Lac) was eventually used for analysis.

Cell-free supernatant obtained from all the production methods was stored at – 20 °C until further use, and all enzymatic procedures were performed at room temperature.

### Enzyme assays

Laccase activity was determined by the oxidation of ABTS^27^ at 28 ± 2 °C. The reaction mixture contained 0.6 ml of 1.6 mM ABTS, 0.6 ml of sodium acetate buffer (pH 5.2) and 0.6 ml of culture filtrate. Distilled water (0.6 ml) without enzyme served as a control. ABTS oxidation was monitored by measuring the increase in absorbance at 420 nm (ε_420_-36,000 M^−1^ cm^−1^). To correct for ABTS oxidation by peroxidases (in media), 0.5 µg/ml catalase was used. One unit of laccase activity was defined as μmoles of ABTS oxidized per minute per ml. Protein concentration was determined by the method of Lowry (1951)^28^ using bovine serum albumin (BSA) as the standard.

### Sectioning of PUF cubes

Polyurethane foam (PUF) cubes inoculated and immobilized with *S. commune* for 20 days were subjected to 0.1 mm micro sectioning using a refrigerated microtome cryostat LEICA CM 1510S. Uninoculated PUF cubes soaked in distilled water served as control. The sections were then observed for fungal colonization using phase-contrast microscopy.

### Preparation of substrate

Paddy straw (PS), finger millet straw (FMS), wheat straw (WS), maize straw (MS), and sorghum stover (SS) were procured from the local market and manually chaffed into lengths of 2 cm. Known weights of the straw were treated by spraying laccase enzyme obtained from different production methods under hydrated conditions. For every 3 g of straw used, 5 ml of enzyme-rich broth was used at a ratio of 3:5 (w/v). Purified laccase was dissolved using Milli-Q water to make up the volume. The enzyme and straw were allowed to react for 24 h at 28 ± 2 °C (room temperature), dried overnight at 60 ± 2 °C, ground to pass through a 1 mm screen, and analyzed for changes in proximate composition and lignin degradation. Straws treated with distilled water without enzyme in the ratio of 3:5 (w/v) served as controls.

### Estimation of cell wall components and cell wall degradation

Proximate principles^29^ and detergent fiber were estimated^30^ in the straws, and the dry matter of control and treated samples was determined after drying the samples at 100 ± 5 °C for 8 h.

### In vitro*,* dry matter digestibility (IVDMD) procedure

In vitro*,* dry matter digestibility (IVDMD) or apparent digestibility was estimated using standard procedures maintaining 39 °C temperature throughout the experimental procedure. Fresh rumen liquor was collected from cannulated animals from the experimental livestock unit of the institute and filtered using a muslin cloth to obtain a clear liquid. A freshly prepared fermentation medium was flushed using CO_2_ (30–40 min) and the reducing agent was added. 100 ml Erlenmeyer flasks containing 0.5 g finely powdered straw samples, rumen liquor, and fermentation medium were sealed with bunsen valves and incubated at 39 °C for 48 h with continuous shaking. After 48 h the contents of each flask were transferred into a 500 ml spoutless beaker containing 100 ml neutral detergent solution. The samples were refluxed for 1 h at 100 °C and then filtered through previously weighed sintered glass crucibles. The samples in the crucibles were washed with hot water to remove the detergent completely and were dried at 100 °C for 24 h before recording the weights. All experiments were performed in three replicates.

### Statistical analysis

Statistical analysis was performed using R statistical software (version 4.1.0)^31^. The data on various nutritional parameters are presented as the mean ± SD (standard deviation) and tabulated. Box plots were designed using ggboxplots, and correlation graphs were plotted using the ggpubr R package^32^. R package ggplot2 was used to create a line graph to see the variations in laccase titre levels across incubation days for different production methods employed^33^. Each component of the graphs graph—axes, scales, colors, objects, etc. was built up sequentially one component at a time. Regression analysis of IVDMD against lignin content governed by each individual production method was performed using the linear regression method to measure the strength of the linear relationship between lignin degradation and in vitro dry matter digestibility^34^. The Geom_smooth function of R with the formula y ~ x was used to analyze the correlation between ADL and IVDMD of five different straws treated with laccase-rich cell-free media.

The coefficients of determination (*R*^2^) were calculated using the formula

$$r = \frac{{\sum {(x - m_{x} )(y - m_{y} )} }}{{\sum {(x - m_{x} )^{2} \sum {(y - m_{y} )^{2} } } }},$$

where ‘mx’ and ‘my’ are the means of x and y variables. The p-value of the correlation was determined using a correlation coefficient table for degrees of freedom: df = n − 2, where n is the number of observations in the x and y variables. For all statistical analyses, significance was declared at P ≤ 0.05 for variables to remain in the model. ANOVA was used to compare the means of ADL and IVDMD of enzyme-treated straws. Further independent t-tests were performed to compare the effect of different methods on each type of straw.

## Results and discussion

Rumen physiology demands the use of both roughages and concentrates in the diet in varying proportions influencing productivity and maintenance costs^35,36^. The low quality of dry roughages necessitates the need for enhancing the nutritive value of the feed using economically favorable delignification methods. Recalcitrant lignin is made up of three monolignols: p-coumaryl, coniferyl, and sinapyl alcohols^37^ and digestion of cell wall fractions of forage in the rumen is incomplete due to the complex links which limit their degradation^38^. White rot fungi are known to be active lignin degraders as they harness a plethora of ligninolytic enzymes and the depolymerization rate by fungi ranges from 20% to nearly 100%, depending on different lignin sources^39^. *S.commune* has been used as a model organism for the production of ligninolytic enzymes^40^ and there has been an increasing interest in exploiting the potential of *S.commune* in various biotechnological processes due to the presence of protein-coding genes like laccases, glycoside hydrolases, lytic polysaccharide monooxygenases (LPMOs) and expansins—like proteins^41^, production of ligninolytic enzymes^14,42,43^, use in mixed cultures^44^. Various types of ligninolytic enzymes are produced by WRF in nature based on the substrate upon which it acts. Laccase, lignin peroxidase (LiP), manganese peroxidase (MnP), versatile peroxidase (VP), and dye-decolorizing peroxidase (DyP) are well-known enzymes for lignin degradation^45^ and take advantage of the electron transfer mechanism to oxidize lignin structure and promote subsequent reactions by giving phenoxy radical or carbon-centered radical intermediates^46^.

Most of the studies using WRF were conducted to a large extent employing SSF. In this study, the native isolate *S. commune* NI-07 was initially grown under solid-state fermentation (SSF) conditions by inoculating the fungus directly onto the lignocellulosic substrate. It produced laccase effectively measuring laccase activity of 304.62 U ml^−1^ on the 5th day, which was relatively higher than that observed for the reference culture *T. versicolor*. However, regardless of SSF appearing to be a viable option, there were dry matter losses as the culture started utilizing the carbohydrate components for carbon source, limiting induction of ligninolytic enzymes, a fact attributed to the fungus shifting its reproductive cycle from asexual to sexual mode. High laccase activity was nevertheless observed during the mycelial growth stage, which dropped down rapidly through fruiting body formation (data not shown). Therefore, the focus was shifted to submerged fermentation methods for laccase production to overcome the limitations of SSF.

**Figure 1:**
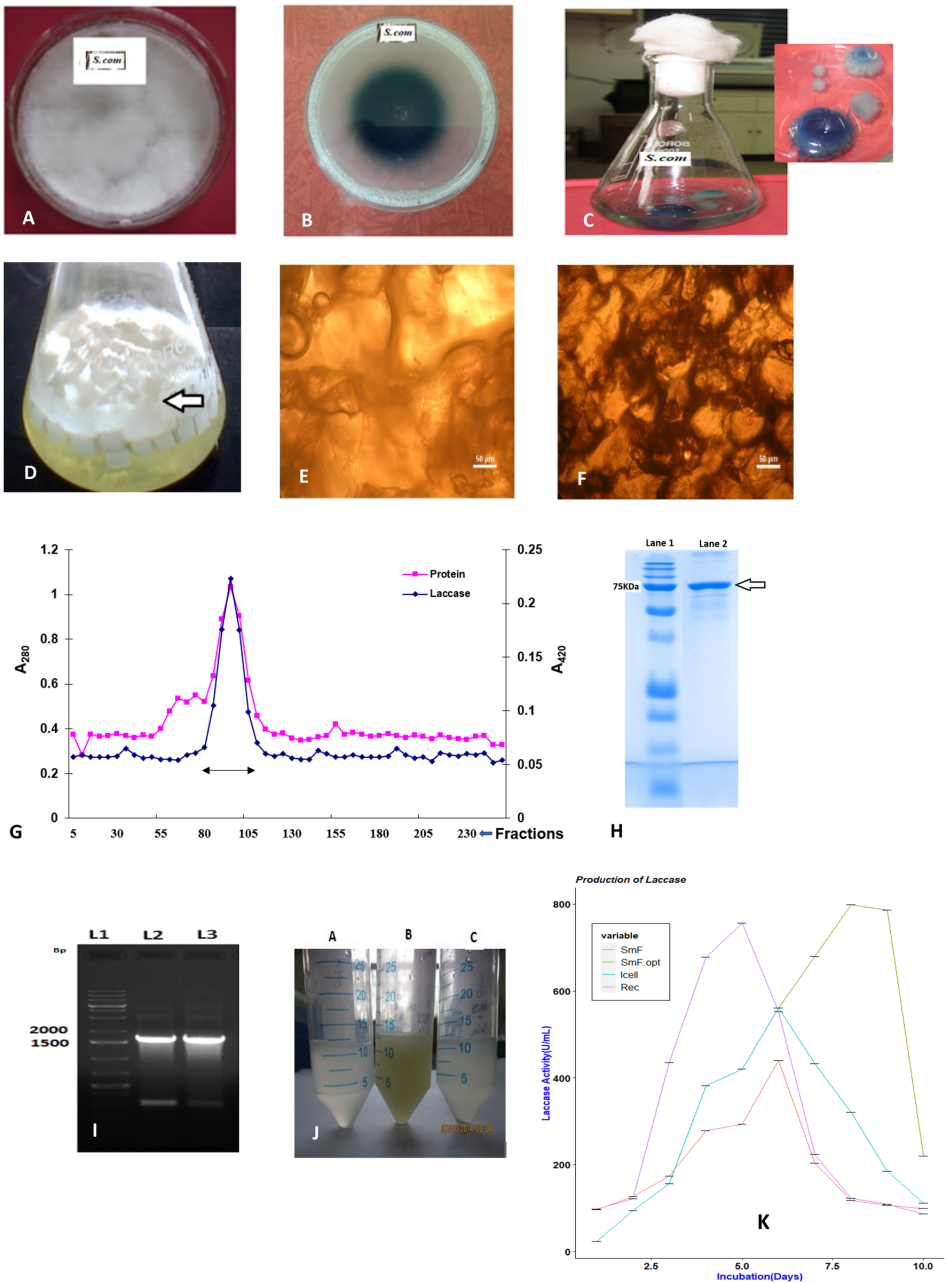
Methods of laccase production using WRF *S. commune* NI-07 strain. Photographs of (**A**) Pure culture of *S. commune* on Malt Extract Agar plate (**B**) *S. commune* grown on Laccase Detection Agar (gL-1 media contained KH_2_PO_4_-1 g; C_2_H_8_N_2_O_4_-0.5 g; MgSO_4_-0.5 g; CaCl_2_-0.01 g; Yeast Extract-0.01 g; CuSO_4_-0.001 g; FeSO_4_-0.001 g; MnSO_4_-0.001 g; 1.6% w/v agar) with 0.1% ABTS as substrate (**C**) Submerged cultivation of *S. commune* in Modified Liquid Basal Medium (LBM). Insert: Close up view of the flask (**D**) Modified LBM with *S. commune* immobilized PUF cubes (**E**,**F**) Phase-contrast micrograph (×40 dry objective, wet mount) showing (**E**) A section of the uninoculated polyurethane foam (PUF) cube (**F**) A section of PUF cube immobilized with *S. commune* (**G**) Elution profile of *S.commune* laccase from Sephadex G-50 gel filtration column (**H**) SDS PAGE of *laccase* obtained through immobilization: Lane 1: Protein standard marker (Biorad Precision Plus), Lane 2: Purified laccase (**I**) Transformed *E. coli* cells analyzed for the presence of pPI9K/Scom-Lac expression cassette (a): Colony PCR of transformed *E. coli* DH5α cells with pPIC9K/Scom-Lac expression cassette. L1-Gene ruler 1 kb plus DNA Ladder, L2&3–5 µl PCR product from two different clones selected on LB/ampicillin plates and loaded on 1% agarose gel (**J**) Laccase expression in methanol induced minimal broth with 0.1 mM CuSO_4_ and 0.5 mM ABTS A-Control GS115 strain without insert; B-Laccase induced strain (GS115/pPIC9K/Scom-lac); C-Sec HSA strain (GS115 Albumin Mut^s^) (**K**) Laccase production using basal salts medium*.* Production (a) under Submerged fermentation (SmF), Submerged fermentation using optimized media (SmF/Opt), Fermentation using immobilized cell (Icell/Lac), Fermentation using recombinant laccase (Rec/Lac. One unit laccase is expressed as the activity of the enzyme that catalyzes the conversion of 1.0 μmole of ABTS min^−1^.

Han et al.^15^ also reported the use of *S. commune* Han 881 to produce laccase in submerged fermentation in mixed cultures accelerating enzyme production. Liquid cultivation of the native isolate *S. commune* (Fig. 1A) produced laccase in the culture media extracellularly and a confirmatory test for laccase activity was made quantitatively by spectroscopic enzymatic analysis. There was an appearance of a green halo around the colony using the ABTS plate test assay (Fig. 1B) confirming the conversion of ABTS to ABTS^+^ radicals in the presence of laccase. Laccase production by *S. commune* under SmF using modified liquid basal solution was reverberated by the overlay of blue color on the mycelium (Fig. 1C), as type I copper on interaction with laccase protein influenced the formation of blue color. Laccase titres were greatly influenced by the production methods employed. The production of laccase under SmF conditions (Fig. 1K) showed maximum laccase activity on the 6th and 7th days in modified BSS (SmF) and the 8th and 9th days in statistically optimized culture media (SmF/Opt).

Immobilization of the native isolate was performed following submerged cultivation as it provides a solution for more cost-effective and economical use of enzymes^47^. Here, *S. commune* cells (Icell/Lac) immobilized using polyurethane foam (PUF) (Fig. 1D) further enhanced laccase production. PUF was used as a supporting platform for adhesion because of its inert nature, excellent dimensional stability, and strength. In the present study, maximum laccase activities were observed on the 6th and 7th days of production. Media-rich in laccase could be recovered easily without compromising cell viability for 9–10 batches. Figure 1E,F shows section micrographs of PUF cubes after 20 days of fungal immobilization (Fig. 1F). Rapid colonization of foam was observed using a phase-contrast microscope when microsections of PUF inoculated with *S. commune* were observed at 40× magnification compared to the control (Fig. 1E), ascertaining adsorption of the fungal mycelia within the matrix. PUF has been used earlier as a support matrix to immobilize fungus. Luke and Burton^48^, immobilized *Neurospora crassa* in a membrane bioreactor to witness the continuous production of enzymes without deactivation for several weeks. Struszczyk-Świta^49^ used PUF-immobilized fungal chitosanase–lipase preparation that was found to have a half-life of 200 days when stored at room temperature. Krishna Prasad et al.^50^ have immobilized *Pleurotus ostreatus* 1084 on PUF cubes and reported increased laccase yields. Reference^51,52^ immobilized *Aspergillus flavus* A5p1 on PUF and showed enhanced RB4 decolorization efficiency. All these reports are in line with our current study and suggest the use of PUF as a suitable matrix for cell immobilization.

The harvested media from immobilization was further used to purify the enzyme. Purification and characterization epitomized the isolate’s superior pH and temperature tolerance ability. Figure 1G shows the elution profile after the Sephadex G-50 size exclusion chromatography. The specific activity of the pure enzyme was 360 U mg^−1^. Gel electrophoresis after concentrating the active fractions from size exclusion chromatography resulted in an apparent molecular weight of about 75,000 Da (Fig. 1H). The results are in agreement with Vantamuri and Kaliwal^53^, where a monomeric protein with a molecular mass of ~ 75 kDa as estimated by non-denaturing polyacrylamide gel electrophoresis was isolated from *Marasmius* species BBKAV79 with greater pH and thermal stability. Irshad and Asgher^54^ purified laccase from *Schyzophyllum commune* IBL-06 and obtained a 63 KDa band on SDS-PAGE. These variations observed in the molecular sizes of laccase obtained from the same organism or across different organisms are because of changes in the glycosylation patterns that occur due to structural and chemical changes of the protein post immobilization. The higher stability observed in the obtained laccase is because immobilization stabilizes the structure of the enzyme and alters the spectral properties both physically and chemically and glycosylation improves the global stability of the protein as well as increases its molecular weight.

The Laccase gene was expressed heterologously in the *Pichia pastoris* GS115 strain for recombinant protein production. The expression cassette containing the optimized laccase gene was transformed into *E. coli* DH5α first for propagation and then transformed into *Pichia*. The transformants from *E. coli*, containing the pPIC9K/Scom-Lac expression cassette with α-factor secretion signal and 3′AOX1 primers were subjected to colony PCR to confirm the presence of insert. Parent plasmid pPIC9K will produce a 195 bp fragment and with a laccase insert of 1520 bp, the amplification product is 1715 bp as evident from the gel photograph corresponding to the primer positions on the vector (Fig. 1I) confirming the successful cloning of pPIC9K with optimized laccase gene. Liquid cultivation of the positive *Pichia* clone using buffered minimal methanol (BMM) decolorized the broth to yellowish-green (Fig. 1J) suggesting proper processing of the signal sequence and extracellular expression of laccase protein was confirmed by ABTS assay. The highly expressing transformant gave a wet cell weight of 0.19 g/10 ml of broth with laccase activity of 344 U ml^−1^ after 5 days of growth at 30 °C. Rec/Lac expressed in the medium contained very little native protein, laccase being the sole enzyme produced with maximum activity on the 5th day (Fig. 1K). Hirai et al.^40^ had also expressed laccase cDNA from white-rot fungus *S. commune* in a transgenic tobacco plant by decreasing the CpG-dinucleotide motif content for phytoremediation of recalcitrant environmental pollutants.

The results obtained in the present study across various production methods, accentuated for 5–7 days of cultivation in liquid cultures for laccase production at 30 °C. It is partially in agreement with the works of Irshad and Asgher (2016) where maximum enzyme activity from *S. commune* was recorded after 192 h using banana stalk under SSF conditions. A pH range between 4 and 5.5 was ideal for laccase production, as it recorded maximum laccase activity in all the production methods employed in that pH range. Many studies on fungi have previously reported the optimal pH range for laccases to be between 3.0 and 6.0^55,56^ and are substrate-dependent. Differential substrate protonation patterns contribute to differences in optimal pH for activity. This is completely in agreement with the results of the current study as well as the study by Nagai et al.^57^, who observed the optimal pH to be 3.0 and 4.0 when the substrates ABTS and o-toluidine were used. Each production step employed greatly increased laccase yields. This could be due to the inherent differential capacity of organisms to synthesize ligninolytic enzymes^58^ under the reaction conditions used.

Crop residues are generally fed to ruminants and form an intricate part of their diets. Previous studies have revealed enhanced ruminant digestibility upon feeding pre-treated crop residues using ligninolytic enzymes of WRF^59^. The present research explored the interaction of laccase enzymes with five different crop residues: paddy straw (PS), finger millet straw (FMS), wheat straw (WS), maize straw (MS), and sorghum stovers (SS). Herein, the influence of various production methods on laccase titre and crop residue oxidation was studied considering parameters such as loss in total dry matter (DM), crude protein (CP), neutral detergent fiber (NDF), acid detergent fiber (ADF) and acid detergent lignin (ADL). Straws were cut into 2 cm lengths and steam sterilized first to provide enough hydration before enzyme treatment, as a hydrated environment creates natural conformation of the enzyme, allowing functional degradation of lignin. Varying concentrations of the enzyme with varying specific activities were obtained when different production methods were employed. Enzyme-rich broth was then sprayed directly onto the straws and mixed well to create a large surface area for enzyme action.

**Figure 2:**
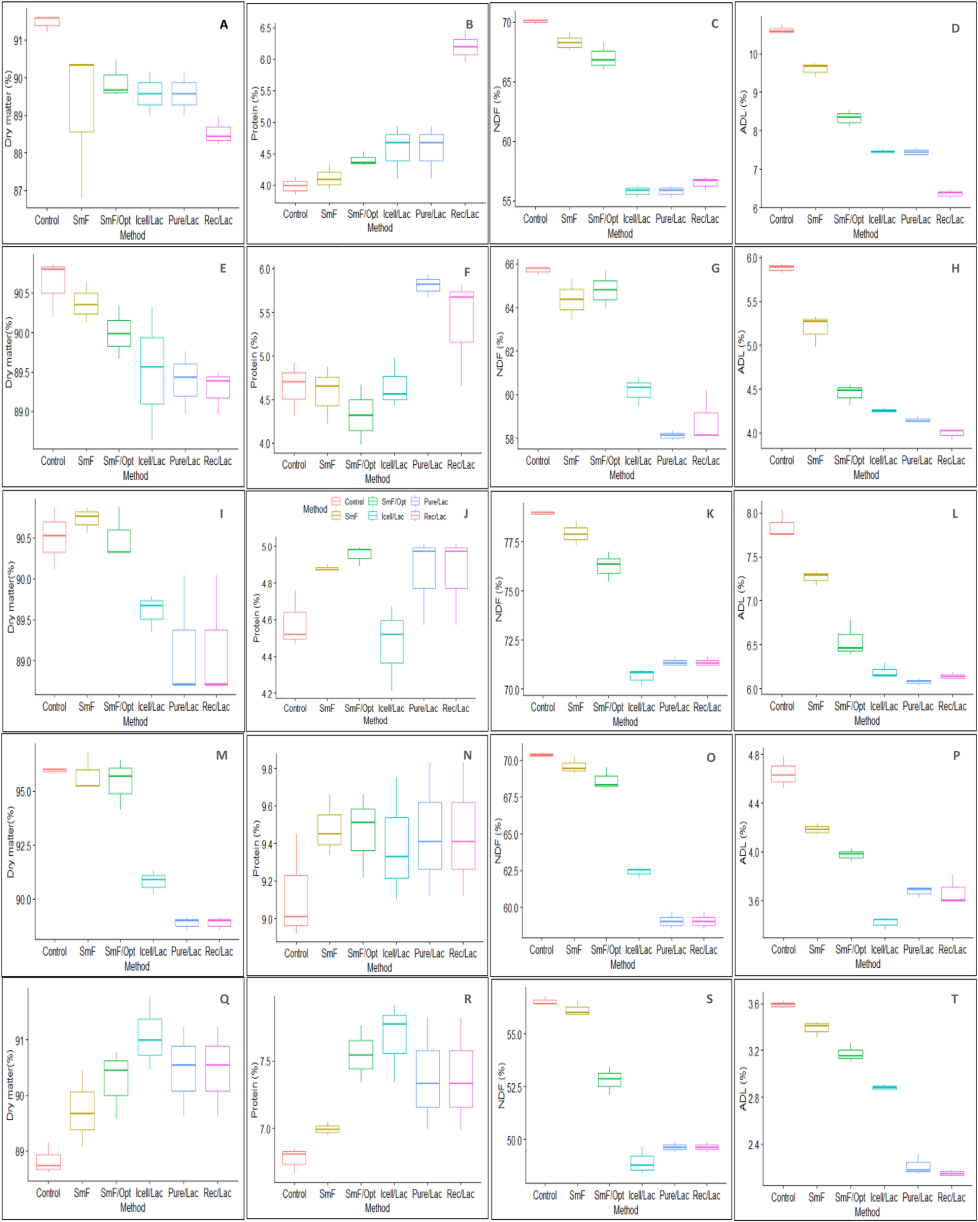
Box and whisker plots showing the distribution of proximate composition of five different crop residues after treatment using laccase enzyme: (**A**–**D**) finger millet (FMS); (**E**–**H**) paddy (PS); (**I**–**L**) wheat (WS); (**M**–**P**) maize (MS) and (**Q**–**T**) sorghum (SS). Labels (**A**,**E**,**I**,**M**,**Q**) represents dry matter (DM)%, (**B**,**F**,**J**,**N**,**R**) represents Protein %, (**C**,**G**,**K**,**O**,**S**) represents Neutral Detergent Fiber (NDF)%, (**D**,**H**,**L**,**P**,**T**) represents Acid Detergent Lignin (ADL)% of FMS, PS, WS, MS and SS respectively. The representative color codes for each production method are as follows Control (pink); laccase from Submerged fermentation, SmF (amber); laccase produced using optimized media in submerged fermentation, SmF/Opt(green); laccase obtained from immobilized cells, Icell/Lac (cyan); purified laccase, Pure/Lac (sky blue) and recombinant laccase, Rec/Lac (Magenta). Bars represent the standard error of means from three replicates.

The proximate composition of each straw treated with laccase obtained from various methods was analyzed. The distribution of parameters in treated straws compared to the control is illustrated using box-and-whisker plots (Fig. 2). A decrease in dry matter (Fig. 2. A,E,I,M,Q) was observed in all the straws (Control > SmF > SmF/Opt > Icell/Lac > Pure/Lac > Rec/Lac), except for SS (Fig. 2Q), where enzyme treatment did not influence dry matter content (control M = 88.83, SD = 0.28; Rec/Lac M = 90.46, SD = 0.81). A linear increase in CP (Fig. 2B,F,J,N,R) content was observed from SmF to Rec/Lac, with Rec/Lac treatment showing higher averages, demonstrating a positive influence of treatment in all the straws studied. This increase in the protein content could be attributed to the addition of the enzyme, which is a protein in nature.

The digestibility of the feed material varies with the type of substrate employed. Different culture methods used to produce laccase and the differences in the physical composition of lignin monomers in each straw based on the level of maturity, growth conditions, and structural variations it carries contributed to the variations in the results obtained. Values of NDF (Fig. 2C,G,K,O,S), ADF (data not presented) and ADL (Fig. 2D,H,L,P,T) showed a steep decrease from control through various methods up until Rec/Lac with marginal difference in values of NDF and ADF for the straws treated with laccase obtained from Pure/Lac and Rec/Lac methods. Decreases in NDF, ADF, and ADL suggested that vegetal cell wall components of the straws were degraded due to laccase treatment, facilitating enhanced digestibility. The results are consistent with earlier reports where different types of dry pastures were treated with 60% enzyme^60^.

There were significant differences in ADL between laccase production from SmF (M = 9.6; SD = 0.19), SmF/Opt (M = 8.32; SD = 0.22) and Rec/Lac (M = 6.35; SD = 0.12) in FMS and SmF (M = 3.39; SD = 0.07), SmF/Opt (M = 3.17; SD = 0.08) and Rec/Lac (M = 2.15; SD = 0.03) in SS when compared to control (M = 10.61; SD = 0.11 and M = 3.59; SD = 0.03 for FMS and SS simultaneously). While PS, WS, and MS showed significant differences in the SmF and SmF/Opt methods, no significant differences were observed between laccase produced from Icell/Lac, Pure/Lac, and Rec/Lac for PS, WS, and MS in terms of lignin content. Differences existed in the p-hydroxyphenyl (H), guaiacyl (G), and syringyl (S) subunits of lignin, and these differences influenced the rate of biodegradability of the straws. Wheat straw contains percent H, G, and S units in a ratio of 5:49:46 ratio of 6:64:30^61,62^ with β-*O*-4´-ether linkages, whereas the H, G, and S units appear in a ratio of 15:45:40 in rice straw. However, an H: G:S ratio of 5:71:24 with higher levels of β-*O*-4′ alkyl-aryl ether linkages with greater guaiacyl lignin was observed by Rosado et al.^63^. Akin et al.^64^ correlated the degradation of guaiacyl monomers in lignin to improve digestibility, while Crestini et al.^65^ observed depolymerization of lignin on the cleavage of the β-*O*-4′ bonds.

ANOVA was conducted to compare the means of ADL and IVDMD of straws treated with laccase. An independent t-test was performed to determine the effect of different production methods on the ADL content and IVDMD of the treated straws (Table 1). Mean values concerning each method for ADL and IVDMD were found to be significantly different (p < 0.001). Treatment demonstrated a gradual decrease in lignin content, with a maximum degradation of 40.1% observed in FMS and SS, followed by PS (32%) and WS (21.7%), with MS (20.9%) showing the least degradation. These observations suggest that crop residue lignin is guaiacyl rich, with β-*O*-4´-linkages dominating the structure. Additionally, cell wall cross-linking impacts biomass saccharification levels. McKinley et al.^66^ observed changes in the hemicellulose-bound *p*-coumarate (pCA) and ferulate (FA) of sorghum stems, showing improved saccharification owing to the presence of a larger proportion of fermentable sugars for access, improving digestibility. The ash content in the straws measures degradation indirectly. Organisms capable of more lignocellulose degradation leave more residual ash. The increase in ash content in all the treated straws compared to the control further validated the results (data not shown). The increase in ash content was similar to the studies conducted by Yasar and Tosun^67^ where the nutritional quality of tomato pomace was improved by *Pleurotus ostreatus* and *Phanerochaete chrysosporium* fermentation.

**Table 1:** Evaluation of ADL and IVDMD of crop residues treated with laccase obtained using different production methods.

Straw	Method	ADL	P value**	IVDMD	P value**
Finger millet	Control	10.61 ± 0.11^a^*	< 0.0001	40.34 ± 0.3^a^	< 0.0001
	SmF	9.6 ± 0.19^b^		45.92 ± 0.75^b^	
	SmF/Opt	8.32 ± 0.22^c^		49.84 ± 1.23^c^	
	Icell/Lac	7.46 ± 0.05^d^		62.37 ± 0.07^d^	
	Pure/Lac	7.44 ± 0.11^d^		62.4 ± 0.11^d^	
	Rec/Lac	6.35 ± 0.12^e^		64.16 ± 0.08^e^	
Paddy	Control	5.88 ± 0.05^a^	< 0.0001	42.4 ± 0.21^a^	< 0.0001
	SmF	5.19 ± 0.18^b^		44.76 ± 1.02^b^	
	SmF/Opt	4.60 ± 0.12^c^		49.5 ± 1.55^c^	
	Icell/Lac	4.25 ± 0.03^d^		56.51 ± 0.21^d^	
	Pure/Lac	4.15 ± 0.03^d^		58.5 ± 0.27^d^	
	Rec/Lac	3.99 ± 0.06^d^		57.84 ± 1.15^d^	
Wheat	Control	7.85 ± 0.16^a^	< 0.0001	43.57 ± 0.09^a^	< 0.0001
	SmF	7.26 ± 0.08^b^		44.94 ± 0.78^b^	
	SmF/Opt	6.54 ± 0.21^c^		49.81 ± 0.82^c^	
	Icell/Lac	6.19 ± 0.09^d^		58.12 ± 0.12^d^	
	Pure/Lac	6.07 ± 0.04^d^		59.4 ± 0.44^d^	
	Rec/Lac	6.14 ± 0.04^d^		59.4 ± 0.44^d^	
Maize	Control	4.64 ± 0.13^a^	< 0.0001	48.84 ± 0.04^a^	< 0.0001
	SmF	4.18 ± 0.05^b^		51.55 ± 1.39^b^	
	SmF/Opt	3.97 ± 0.05^c^		54.14 ± 0.58^c^	
	Icell/Lac	3.42 ± 0.05^d^		56.73 ± 0.23^d^	
	Pure/Lac	3.67 ± 0.05^d^		55.71 ± 0.17^d^	
	Rec/Lac	3.67 ± 0.12^d^		55.71 ± 0.17^d^	
Sorghum	Control	3.59 ± 0.03^a^	< 0.0001	60.34 ± 0.08^a^	< 0.0001
	SmF	3.39 ± 0.07^b^		62.25 ± 1.3^b^	
	SmF/Opt	3.17 ± 0.08^c^		65.18 ± 0.25^c^	
	Icell/Lac	2.88 ± 0.03^d^		68.19 ± 0.21^d^	
	Pure/Lac	2.22 ± 0.09^e^		69.63 ± 0.17^d^	
	Rec/Lac	2.15 ± 0.03^e^		69.63 ± 0.17^d^	

Variables with different superscripts within groups were found to be statistically significant.

(p- value < 0.05 for t-test); ** statically significant if p- value < 0.05 (for ANOVA).

Substrate particle size also influenced treatment significantly, as it created a large surface-to-volume ratio, thereby providing access to carbohydrate components. In the present study, crop residues were cut into 2 cm lengths before treatment. This is in agreement with earlier studies from Salvachua et al.^68^, where the digestibility of *Irpex lacteus*-treated wheat straw cultures improved significantly with a reduction in particle size.

IVDMD (t-test) showed a significant difference in treated straws for the SmF, SmF/Opt, and Icell/Lac methods, while no significant difference was observed for Icell/Lac, Pure/Lac, and Rec/Lac in any of the straws used (Table 1). Maximum digestibility was observed in FMS, recording 59% enhancement, while PS and WS showed 36% enhancement in digestibility. Increases in IVDMD of 14.44% and 15.83% were recorded in MS and SS, respectively. Although SS showed maximum lignin degradation, IVDMD was very low compared to other straw samples and can be attributed to the genetic variability it carries. Si et al.^69^ isolated the laccase gene from *Agrobacterium* sp. S5-1 in soil humus and heterologously expressed in *Escherichia coli*. The authors observed that laccase produced significantly (*p* < 0.05) increased dry matter digestibility of maize straw from 23.44 to 27.96% and from 29.53 to 37.10% after 8 or 24 h of digestion. Many researchers have reported similar observations for straw treatment under SSF conditions. Results obtained in the present study are in agreement with the experiments conducted by Datsomor et al.^70^ where pretreatment of rice straw using *P. ostreatus* showed a marked decrease in acid detergent lignin when compared to control. The IVDMD and the total VFA concerning *P*. *ostreatus* were markedly higher than both the control and the other treatments. Similar results were obtained by Sufyan et al.^71^ where the nutritional value and digestibility of wheat straw, rice straw, and corn cob using *Pleurotus* species improved.

**Figure 3:**
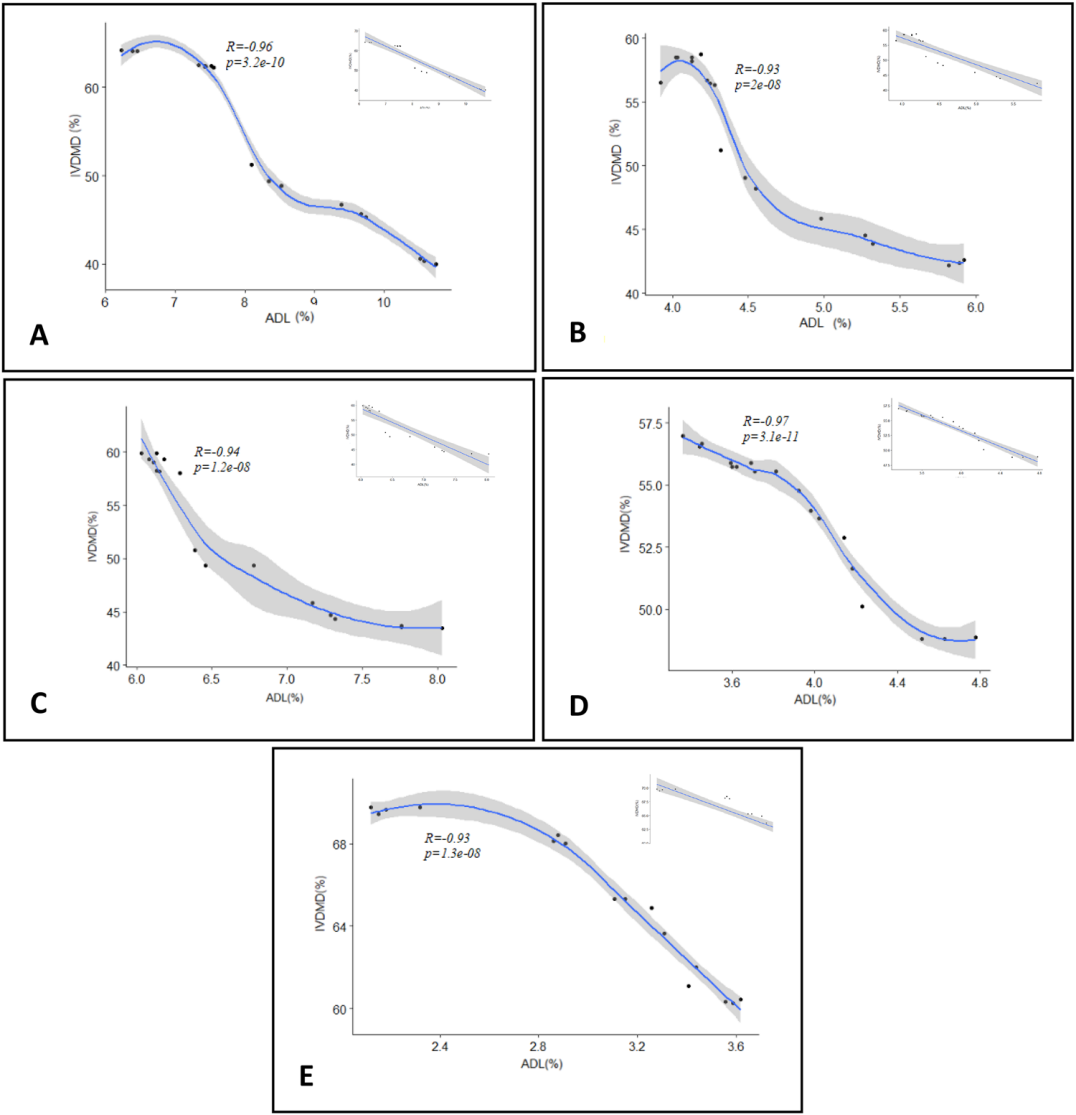
Correlation of acid detergent lignin (% ADL) to in vitro digestibility of dry matter (% IVDMD) of laccase treated crop residues (**A**) finger millet (**B**) paddy (**C**) wheat (**D**) maize and (**E**) sorghum Insert: Linear Regression graphs to generate R values.

Comparative analysis of the correlation between lignin content and dry matter digestibility on treatment with laccases (Fig. 3) showed a positive effect on delignification irrespective of the production method employed. A strong negative correlation was observed between enhancement in digestibility and lignin degradation in all the production methods used, with Icell/Lac, Pure/Lac, and Rec/Lac showing maximum degradation and increased digestibility.

Linear regression graphs used to generate ‘r’ values (Fig. 3, Insert) showed a very strong negative correlation for both FMS (Fig. 3A) and MS (Fig. 3D) (r = − 0.9595558 and − 0.9698371, respectively), while PS (Fig. 3B) (r = − 0.9315393), WS (Fig. 3C) (r = − 0.9356731) and SS (Fig. 3E) (r = − 0.9348795) gave a strong negative correlation (p < 0.0001). Laccases from Icell/Lac, Pure/Lac, and Rec/Lac showed maximum lignin degradation with increased digestibility while having similar effects in all the straws studied with Pure/Lac and Rec/Lac. The linear increase obtained in the present study in IVDMD of five straws upon treatment with laccase is in keeping with earlier reports. Ravichandran et al.^63^ used versatile peroxidase enzyme produced through submerged fermentation for treating finger millet straw and recorded a 14% and 16% increase in digestibility. A maximum improvement of 20% in IVDMD was obtained upon treatment of different straws with purified lignin peroxidase by Thammaiah et al.^22^. In the current study, lignin degradation and digestibility in maize straw were more effective when Icell/Lac was used compared to Pure/Lac and Rec/Lac. Crop residue lignin of maize straw is known to be syringyl rich with beta aryl ether linkages, as confirmed by 2D NMR studies^72^. Syringyl propane units predominantly are acid-soluble. However, the pattern of degradation and digestibility differed in all other straws, which are rich in guaiacyl units. This confirms the fact that the guaiacyl–syringyl ratio plays a major role in influencing the degradation pattern.

Extracellular enzymes are generally released in minute quantities and research favors the use of optimization strategies to improve enzyme production^73^. Many workers have emphasized the need for developing cost-effective environmentally safe strategies to ameliorate the nutritive value of the feed for better productivity using WRF. Zhang et al.^74^, highlighted the potential of WRF to use enzymes for higher fiber digestion with low price and environmentally friendly properties. Zhu et al.^43^ pointed out the use of crude ligninolytic enzyme extracts to be a novel approach that can help develop a cost-effective and environmentally acceptable technology, as they present several additional advantages over the use of purified enzymes. The presence of proteins, mediators, or other factors in the medium may stabilize crude enzymes and mediate the action of these enzymes^75^.

In the present study, employing immobilized, highly stable laccase enzyme-rich media, to enhance in vitro digestibility of the tested crop residues parallels the aforementioned research.

Digestibility values obtained upon the use of Icell/Lac, Pure/Lac, and Rec/Lac after treatment did not demonstrate remarkable variation proving that the former method was equally effective with the latter two. This would save the additional expenses and labor involved in the purification of laccases, as immobilized laccase can be repeatedly produced at a very low input cost along with the fact of remaining a relatively easy, safe, and environmentally friendly technology.

## Conclusion

Ruminant livestock is endowed with the inherent ability to use crop *residues* as feeds. The enormous potential of laccase for several applications necessitated the need for newer hyper-producing strains to overcome the limitations of ruminant digestibility by acting on the lignin polymer selectively. Laccase produced employing solid-state fermentation (SSF) of crop residues results in severe losses in organic matter and is not feasible in the context of ruminant feeding. Laccase enzyme produced using submerged fermentation batch culture is a very convenient nevertheless expensive process. The results of the current study are extremely promising evincing that delignification of all tested crop residues employing immobilized laccase was the most effective and an extremely lucrative technology for potential use in enhancing ruminant digestibility. It also exhibits immense scope in biotechnological and industrial applications. Pilot-scale studies for large-scale production of laccase enzyme through immobilization corroborated with in vivo feeding trials in ruminants are however warranted and would add to confirming the finding of the current study.

## Supplementary Information

Supplementary Information.
