# Supplementary figures and images for: Biological depolymerization of lignin using laccase harvested from the autochthonous fungus Schizophyllum commune employing various production methods and its efficacy in augmenting in vitro digestibility in ruminants
Source: Sci Rep. 2022 Jul 1;12:11170. doi: 10.1038/s41598-022-15211-9 (PMC9249777; doi:10.1038/s41598-022-15211-9)

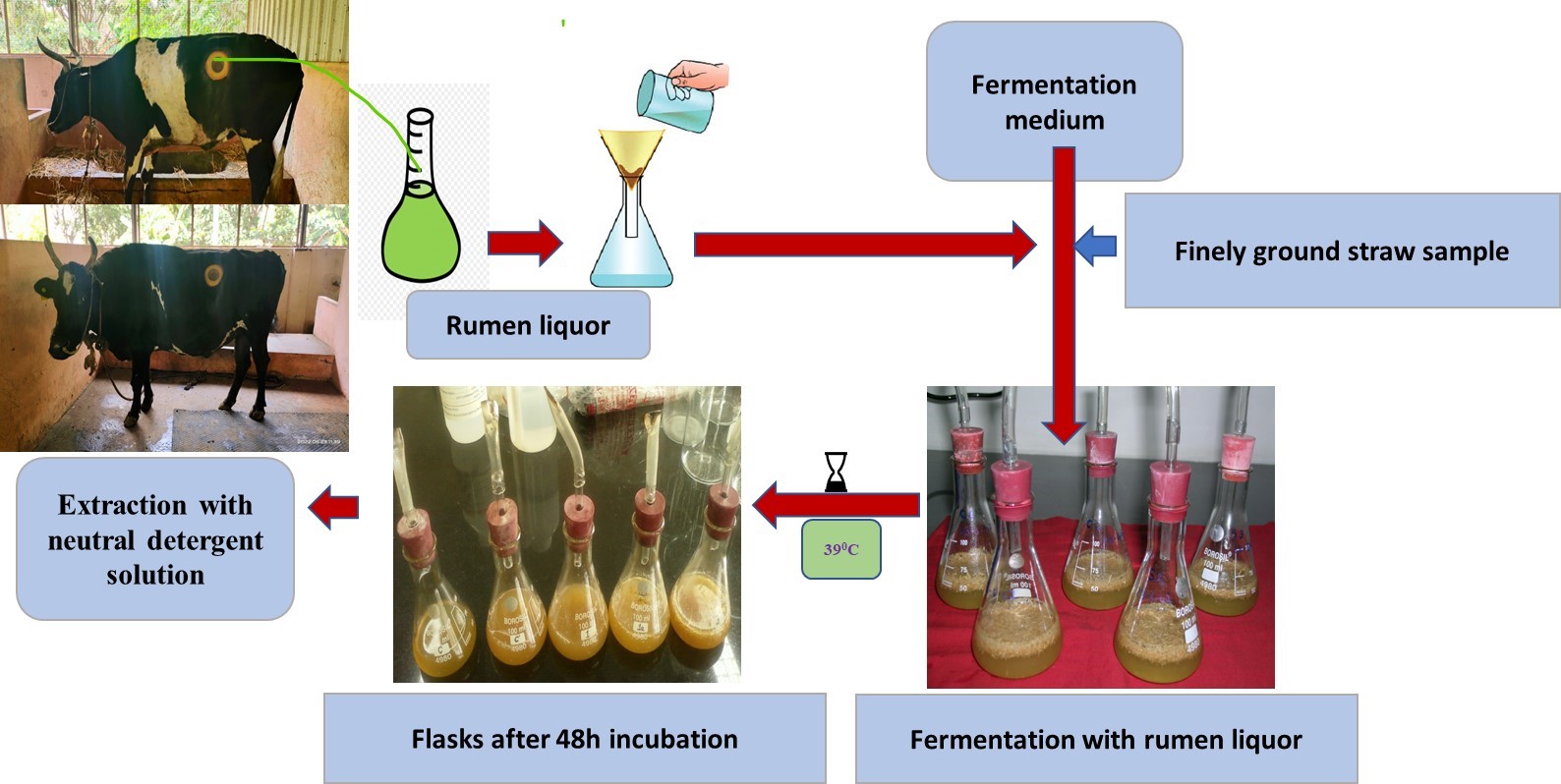

Supplement: Supplementary file 1 — Supplementary Information. [file 41598_2022_15211_MOESM1_ESM.jpg]
